# RETRACTION: Preventive but Not Curative Efficacy of Celecoxib on Bladder Carcinogenesis in a Rat Model

**DOI:** 10.1155/mi/9875382

**Published:** 2026-05-20

**Authors:** Mediators of Inflammation

RETRACTION: J. Sereno, B. Parada, F. Reis, et al., “Preventive but Not Curative Efficacy of Celecoxib on Bladder Carcinogenesis in a Rat Model,” *Mediators of Inflammation*, 2010, 380937, https://doi.org/10.1155/2010/380937.

The above article, published online on 13 February 2011 in Wiley Online Library (wileyonlinelibrary.com), has been retracted by agreement between the journal Editor‐in‐Chief, Anshu Agrawal; and John Wiley & Sons Ltd. The retraction has been agreed due to concerns identified with the figures, both directly and on PubPeer [1]. Further investigation by the Publisher noted additional concerns. To summarize:

• In Figure 2, Panels B and C2 appear identical to Panels B and C2 in Figure 2 of [2]
• In Figure 2, Panels A and B appear identical to Panels A and B in Figure 3 of [3]
• In Figure 2, Panel B appears identical to panel A in Figure 2 of [4], after rotation.

After assessment of the concerns and the authors’ response by the editorial board, the results and conclusions can no longer be considered reliable, and the article is therefore retracted.

The authors disagree with the retraction.
